# Reclassification of obstetric antiphospholipid syndrome under the 2023 ACR/EULAR criteria: risk of under-recognition in patients with non-criteria antibodies and clinical implications

**DOI:** 10.3389/fmed.2026.1929786

**Published:** 2026-10-02

**Authors:** Tingwei Sheng, Yuqi Tang, Zhengwei Tan, Ling Liu, Qiaoding Dai

**Affiliations:** 1 Department of Encephalopathy, Hubei Provincial Hospital of Traditional Chinese Medicine, Wuhan, China; 2 Department of Rheumatology and Immunology, The First Affiliated Hospital of Zhejiang Chinese Medical University, Hangzhou, China; 3 Department of Hematology, The First Affiliated Hospital of Zhejiang Chinese Medical University, Hangzhou, China

**Keywords:** 2023 ACR/EULAR, classification criteria, non-criteria antibodies, obstetric antiphospholipid syndrome (OAPS), pregnancy complications

## Abstract

**Objective:**

To characterize the clinical, serologic, and treatment features of patients with obstetric antiphospholipid syndrome (OAPS), determine the proportion meeting the laboratory domain of the 2023 American College of Rheumatology/European Alliance of Associations for Rheumatology (ACR/EULAR) classification criteria, and examine patients identified through non-criteria antibodies.

**Methods:**

We retrospectively reviewed consecutive women with a prior clinical diagnosis of OAPS at our center and assigned de-identified codes P1-P45. Of the 45 patients, 41 had sufficiently complete historical serologic documentation for laboratory-domain reclassification; P10, P17, P34 and P42 were retained in the descriptive cohort but treated as not assessable in the evaluable-case analysis. Two investigators independently scored each evaluable patient across the six clinical domains and two laboratory domains; classification required a cumulative score of ≥3 in both the clinical and laboratory domains. For patients with concomitant systemic lupus erythematosus (SLE), obstetric or hematologic manifestations attributable to SLE were excluded from APS scoring.

**Results:**

A total of 45 patients were included, all female, with a mean age of 33.7 years (43 primary APS, 2 SLE-associated). Thirty-two patients (71.1%) had a history of pregnancy loss, and 20 had recurrent miscarriage. Anticardiolipin (aCL) IgG and IgM were positive in 9 and 14 patients, respectively; anti-*β*2-glycoprotein I (anti-*β*2GPI) IgG and IgM were positive in 2 and 16 patients, respectively; lupus anticoagulant (LAC) was positive in 2 patients. Fourteen patients (31.1%) carried non-criteria antibodies, including 7 with non-criteria antibodies only. Among the 41 patients with evaluable serologic data, 7/41 (17.1%) definitively met the ≥3-point laboratory threshold and 11/41 (26.8%) met it under the upper-bound assumptions. In the conservative sensitivity analysis, which counted P10, P17, P34 and P42 as below threshold, the corresponding estimates were 7/45 (15.6%) and 11/45 (24.4%). Patients with isolated IgM positivity, IgA-only antibodies, single-positive LAC, or non-criteria antibodies alone did not meet the threshold. An exploratory comparison found no statistically significant difference in documented standardized intervention between live-birth and further-loss cases [28/36 [77.8%] vs 6/9 [66.7%]; two-sided Fisher's exact *p* = 0.666].

**Conclusion:**

In this single-center retrospective series of 45 patients, many patients were identified through non-criteria antibodies or low-weight serologic profiles and therefore did not meet the 2023 laboratory-domain threshold. Because of the small sample, these findings are descriptive and hypothesis-generating and should not be generalized. Nevertheless, they support using the 2023 criteria primarily to define homogeneous research cohorts rather than as a stand-alone basis for diagnosis or treatment decisions. Whether the obstetric phenotype requires a separate classification approach remains to be determined.

## 1 Introduction

Antiphospholipid syndrome (APS) is an autoimmune disease characterized by arterial, venous, or microvascular thrombosis and/or pathological pregnancy in the setting of persistently positive antiphospholipid antibodies (aPL) (1–3). When the obstetric complications predominate and thrombosis is not prominent, the condition is clinically termed obstetric APS (OAPS), a treatable cause of adverse pregnancy outcome in women of reproductive age (4–6).

APS classification has long relied on the revised 2006 Sydney criteria, which require one qualifying clinical event plus persistently positive antiphospholipid antibodies (aPL): lupus anticoagulant (LAC), or medium-to-high-titer immunoglobulin G (IgG) or immunoglobulin M (IgM) anticardiolipin (aCL) or anti-β2-glycoprotein I (anti-β2GPI) antibodies, confirmed on two occasions at least 12 weeks apart (1). In 2023, the American College of Rheumatology (ACR) and European Alliance of Associations for Rheumatology (EULAR) introduced a weighted scoring system: an entry criterion (at least one positive aPL result within the preceding 3 years) is followed by scoring across six clinical domains and two laboratory domains, with classification requiring a cumulative score of ≥3 in both the clinical and laboratory domains. In the validation cohort, the new criteria increased specificity from 86% to 99%, while sensitivity fell from 99% to 84% (7).

The gain in specificity comes at the cost of sensitivity, and this cost falls disproportionately on the obstetric phenotype: recurrent early miscarriage and fetal loss receive comparatively low scores in the obstetric domain; medium-to-high-titer IgM is assigned only 1 point and cannot alone reach the laboratory threshold; solid-phase assays now use a uniform 40-unit cutoff rather than each laboratory's own 99th percentile; and IgA and various non-criteria antibodies receive no points at all. Several obstetric cohorts have shown that, after reclassification under the new criteria, only about one-fifth to one-third of previously diagnosed patients remain classifiable, with the loss of the obstetric phenotype particularly marked among Chinese patients (8–10). On this basis, we retrospectively reviewed the clinical, antibody, and treatment data of OAPS patients at our center, rescored each case according to the 2023 criteria, and, in conjunction with a literature review, analyzed which patients are most likely to be missed.

This loss of sensitivity has direct clinical consequences. Because OAPS is a treatable cause of adverse pregnancy outcomes, patients who fall outside the new criteria may not be offered standard antithrombotic and immunomodulatory therapy (low-dose aspirin, low-molecular-weight heparin, and hydroxychloroquine), or established therapy may be withdrawn. Such undertreatment may lead to preventable recurrent miscarriage, fetal loss, pre-eclampsia, placental insufficiency, fetal growth restriction, and preterm birth, as well as increased maternal thrombotic risk. Identifying the patients most likely to be missed is therefore of immediate clinical importance.

## 2 Materials and methods

### 2.1 Study population and design

This was a single-center, retrospective case series combined with a literature review. Consecutive women with a prior clinical diagnosis of OAPS at our center were included in the descriptive cohort; criterion-based laboratory reclassification was restricted to patients with sufficient historical serologic documentation. Patients with severely incomplete data precluding assessment of the basic phenotype, or whose obstetric events could be fully explained by another clear etiology, were excluded. All cases were coded P1-P45; no names or medical record numbers appear in the text. Recurrent miscarriage was defined as ≥2 pregnancy losses, in accordance with the 2023 ESHRE recurrent pregnancy loss guideline (26); this descriptive definition is distinct from the obstetric thresholds applied for classification (the 2006 Sydney and 2023 ACR/EULAR obstetric domains), which were scored separately for each case. For P10 and P17, no positive aPL result could be retrieved from the available records. Their serologic data were therefore classified as incomplete, rather than antibody-negative, and their 2023 laboratory-domain status was considered not assessable. They were excluded from the evaluable-case laboratory-domain analysis and included only in a conservative sensitivity analysis that counted them as not reaching the threshold. No supplementary testing was performed because this retrospective analysis relied on the available historical records; contemporary testing could not verify serologic status at the time of the original obstetric events.

### 2.2 Data collection

Data collected included age, APS subtype (primary or SLE-associated), pregnancy history, comorbidities (Evans syndrome, secondary immune thrombocytopenia, prior syphilis, etc.), laboratory parameters [white blood cell count, hemoglobin, platelet count, D-dimer, activated partial thromboplastin time (APTT)], imaging assessment for thrombosis, the complete antibody profile (criteria and non-criteria aPL, including titer and isotype), and medications.

### 2.3 Antibody testing

aCL and anti-β2GPI antibodies (IgG/IgM; IgA where tested) were measured by chemiluminescence immunoassay (CLIA) on an iFlash 3000-C analyzer (YHLO Biotech, Shenzhen, China), with positivity defined at ≥20 CU according to the manufacturer's cutoff. For 2023-criteria scoring, medium- and high-titer thresholds were defined as 40–79 and ≥80 units, respectively. LAC was assessed using the dilute Russell's viper venom time (dRVVT) with a confirmatory assay, following ISTH guidance. Non-criteria antibodies (anti-phosphatidic acid, anti-phosphatidylethanolamine, anti-phosphatidylglycerol, anti-phosphatidylinositol, anti-annexin V, and anti-prothrombin/anti-phosphatidylserine-prothrombin) were measured by enzyme-linked immunosorbent assay (ELISA) using commercial kits (EUROIMMUN AG and INOVA Diagnostics, San Diego, CA, USA) according to the manufacturers' reference cutoffs. Where assay details for retrospectively obtained results could not be recovered, this is acknowledged as a limitation. Detailed assay platforms and assay-specific cutoffs are summarized in Supplementary Table S1. Positivity was defined using the cutoff recommended by each manufacturer for the corresponding assay, rather than a single uniform cross-platform threshold. Lot-specific information and between-lot quality-control records were not available for all retrospectively retrieved results; therefore, batch-to-batch variation could not be quantified.

### 2.4 Reclassification method

Two investigators independently scored each patient across the six clinical domains and two laboratory domains of the 2023 criteria; discrepancies were resolved by discussion. Classification under the new criteria required a cumulative score of ≥3 in both the clinical and laboratory domains. In the solid-phase domain, only medium (40–79 units) or high (≥80 units) positive IgG/IgM anticardiolipin or anti-β2GPI antibodies were scored; IgA isotype and non-criteria antibodies were not scored. Lupus anticoagulant was scored according to whether it was positive on a single occasion or persistently, following the International Society on Thrombosis and Haemostasis (ISTH) position on laboratory criteria for APS (11). For patients with concomitant SLE, obstetric or hematologic manifestations attributable to SLE itself were excluded from the APS clinical domain.

### 2.5 Handling of missing data and statistical analysis

A conservative approach was applied: results for which persistent positivity could not be confirmed, or for which quantitative titers were unavailable, were not counted as meeting criteria in the primary analysis. A separate upper-bound analysis assumed that all borderline results were persistently positive, providing a possible range for the reclassification proportion. Missing or unavailable serologic results were not imputed as negative. The primary laboratory-domain analysis was restricted to evaluable cases; a conservative sensitivity analysis additionally counted non-assessable cases as not reaching the laboratory-domain threshold. Normality of continuous variables was assessed with the Shapiro–Wilk test using IBM SPSS Statistics version 26.0 (IBM Corp., Armonk, NY, USA). Normally distributed variables are presented as mean (standard deviation) and non-normally distributed variables as median (interquartile range); categorical variables are presented as number (percentage); for an exploratory outcome-stratified analysis, pregnancy outcome was categorized as successful (live birth) or unsuccessful (further pregnancy loss), and the proportion receiving a documented standardized pharmacologic intervention at our center was compared using a two-sided Fisher's exact test. Because the unsuccessful group was small (*n* = 9), multidrug regimens overlapped, and cells were sparse, treatment-specific tests were not considered reliable and all comparisons were interpreted as exploratory.

## 3 Results

### 3.1 General characteristics

All 45 patients were female, with a mean age of 33.7 years (range 23–55); 43 had primary APS and 2 had SLE-associated APS. Thirty-two patients (71.1%) had a history of pregnancy loss, and 20 (44.4%) had recurrent miscarriage (≥2 losses), with a maximum of 6 miscarriages in one patient. Twenty patients presented or were managed during the index pregnancy. One patient each had concomitant Evans syndrome, six had secondary immune thrombocytopenia, and 2 had a history of syphilis (Table 1).

**Table 1:** Baseline and clinical characteristics (*n* = 45).

Characteristic	All (*n* = 45)	Primary (*n* = 43)	SLE-associated (*n* = 2)
Female	45 (100%)	43	2
Age, years, mean (range)	33.7 (23–55)	—	33
History of pregnancy loss (≥1)	32 (71.1%)	32	0
Recurrent miscarriage (≥2)	20 (44.4%)	20	0
Managed during index pregnancy	20 (of 28)	20	0
Evans syndrome	1 (2.2%)	0	1
Secondary immune thrombocytopenia	6 (13%)	6	0
Prior syphilis	2 (4.4%)	2	0
Thrombocytopenia (PLT < 100)	9 (20%)	8	1
Anemia (Hb < 110)	9 (20%)	8	1
Confirmed thrombosis (imaging)	0	0	0

Hb, hemoglobin; PLT, platelet count; SLE, systemic lupus erythematosus.

### 3.2 Clinical and laboratory characteristics

Thrombocytopenia (<100 × 10⁹/L) was present in 9 patients (20%), with a nadir of 10 × 10⁹/L; anemia (hemoglobin <110 g/L) was present in 9 patients (20%). Following Shapiro–Wilk testing, non-normally distributed variables are reported as median (IQR) and normally distributed variables as mean ± SD: white blood cell count 9.9 (8.1, 11.8) × 10⁹/L, hemoglobin 125 (112, 134) g/L, platelet count 184.4 ± 80.3 × 10⁹/L, D-dimer 0.34 (0.24, 0.89) mg/L fibrinogen equivalent units (FEU), and APTT 24.6 (23.4, 28.2) s (*n* = 41). Imaging did not identify confirmed arterial or venous thrombosis in any patient.

### 3.3 Pregnancy outcomes

Among the 45 patients included, treatment records showed that 34 (75.6%) received standardized pharmacologic intervention at our center after diagnosis, predominantly combinations of low-dose aspirin, heparin, and hydroxychloroquine. On follow-up, the overall live-birth rate was 80% (36/45); the 36 live births occurred at a mean gestational age of 37.3 ± 2.0 weeks, with a mean birth weight of 2,937 ± 479 g. Preterm birth (<37 weeks) occurred in 7 patients (15.6%). Regarding maternal and fetal complications, pre-eclampsia/placental insufficiency occurred in 5 patients (11.1%) and fetal growth restriction (FGR) in 5 patients (11.1%). Nine patients (20.0%) experienced pregnancy loss during follow-up (Table 2).

**Table 2:** Pregnancy outcomes (*n* = 45).

Characteristic	All (*n* = 45)	Primary (*n* = 43)	SLE-associated (*n* = 2)
Live birth, *n* (%)	36 (80%)	35 (81.4%)	1 (50%)
Pregnancy loss during follow-up, *n* (%)	9 (20%)	8 (18.6%)	1 (50%)
Gestational age at delivery, weeks, mean ± SD	37.3 ± 2.0	—	—
Birth weight, *g*, mean ± SD	2937 ± 479	—	—
Preterm birth (< 37 weeks), *n* (%)	7 (15.6%)	7 (16.3%)	0
Pre-eclampsia/placental insufficiency, *n* (%)	5 (11.1%)	4 (9.3%)	1 (50%）
Fetal growth restriction (FGR), *n* (%)	5 (11.1%)	5 (11.6%)	0

Gestational age and birth weight are summarized for all live births (*n* = 36); subgroup values were not computed.

FGR, fetal growth restriction; SD, standard deviation; SLE, systemic lupus erythematosus.

For an exploratory outcome-stratified analysis, successful pregnancy was defined as live birth (*n* = 36) and unsuccessful pregnancy as pregnancy loss during follow-up (*n* = 9). A documented standardized pharmacologic intervention at our center was recorded in 28/36 (77.8%) successful pregnancies and 6/9 (66.7%) unsuccessful pregnancies; the difference was not statistically significant (two-sided Fisher's exact *p* = 0.666). Correspondingly, live birth occurred in 28/34 (82.4%) patients with a documented standardized regimen and 8/11 (72.7%) without such a regimen. Because the unsuccessful group was small and treatment intensity was likely influenced by baseline risk, this comparison is exploratory and does not estimate a causal treatment effect. Drug-specific comparisons were not considered reliable because multidrug regimens overlapped and cell counts were sparse.

### 3.4 Antibody profile

Criteria antibodies: aCL IgG was positive in 9 patients (20%) and IgM in 14 patients (31.1%); anti-β2GPI IgG was positive in 2 patients (4.4%) and IgM in 16 patients (35.6%); LAC was positive in 2 patients (4.4%; dRVVT, 1.30). Non-criteria antibodies and isotypes were common: anti-β2GPI IgA was present in 5 patients, and 14 patients (31.1%) carried non-criteria antibodies, including anti-phosphatidic acid, anti-phosphatidylethanolamine, anti-phosphatidylglycerol, anti-phosphatidylinositol, anti-annexin V, or anti-prothrombin antibodies. P11, P13, P15, P22, P27, P35 and P37 had non-criteria antibodies only; P10, P17, P34 and P42 had incomplete historical serologic documentation and were not classified as antibody-negative (Table 3).

**Table 3:** Antibody spectrum and laboratory data (*n* = 45).

Item	All (*n* = 45)	Positive cases	Notes
Criteria antibodies			
Anticardiolipin (aCL) IgG	9 (20%)	P1, P3, P19, P20, P24, P28, P29,P39, P44	P3 102.32, P20 162.00), P24 161.81,P29 91.46and P39 150.17(high titer); P19 35.21and P44 28.19 (<40 units); P1, P28 positive only
aCL IgM	14 (31.1%)	P2, P3, P8, P9, P14, P16，P21, P23, P25, P26, P30, P36, P40, P43	P3 155.28, high titer
Anti-*β*2-glycoprotein I (anti-β2GPI) IgG	2 (4.4%)	P4, P33	46.03 (moderate)
anti-β2GPI IgM	16 (35.6%)	P3, P5, P6, P8, P14, P16, P18, P21, P23, P25, P26, P30, P36, P40, P41, P45	P3 158.17, high titer
Lupus anticoagulant (LAC)	2 (4.4%)	P7, P32	dRVVT 1.30; P8/P9 negative; P10 negative on the available test, but historical aPL documentation was incomplete
Non-criteria antibodies and isotypes			
anti-β2GPI IgA (non-criteria isotype)	5 (11.1%)	P7, P12, P31, P32, P38	P7 21.96; P12 27.54.P7 and P32 also had LAC positivity; P12, P31, and P38 had anti-β2GPI IgA positivity only.
Non-criteria antibodies only	7 (15.6%)	P11, P13, P15, P22, P27, P35, P37	Anti-phosphatidic acid, anti-phosphatidylethanolamine, anti-annexin V, or aPS/PT
Any non-criteria antibody	14 (31.1%)	P6, P8, P9, P11, P13, P15, P21, P22, P25, P27, P30, P35, P37, P40	Phosphatidic acid, phosphatidylethanolamine, phosphatidylglycerol, phosphatidylinositol, annexin V, or prothrombin-related antibodies
Serologic documentation incomplete	4 (8.9%)	P10, P17, P34, P42	No retrievable historical positive aPL report; laboratory-domain status not assessable
Laboratory data			
White blood cells (10⁹/L), median (IQR)	9.9 (8.1, 11.8)	—	Elevated in P2 after treatment
Hemoglobin (g/L), median (IQR)	125 (112, 134)	—	
Platelet count (10⁹/L), mean ± SD	184.4 ± 80.3	—	
D-dimer (mg/L FEU), median (IQR)	0.34 (0.24, 0.89)	—	
APTT (s), median (IQR)	24.6 (23.4, 28.2)	—	*n* = 41

Missing or unavailable serologic results were not imputed as negative. P10, P17, P34 and P42 were classified as not assessable. Antibody percentages represent documented positives in the full descriptive cohort.

aCL, anticardiolipin; anti-β2GPI, anti-β2-glycoprotein I; aPL, antiphospholipid antibody; APTT, activated partial thromboplastin time; dRVVT, dilute Russell's viper venom time; FEU, fibrinogen equivalent units; IQR, interquartile range; LAC, lupus anticoagulant; SD, standard deviation.

### 3.5 Treatment

Low-dose aspirin was used in 23 patients (51.1%), heparin in 34 (75.6%), hydroxychloroquine in 29 (64.4%), and glucocorticoids in 18 (40%); intravenous immunoglobulin was used in 4 patients, and cyclophosphamide and cyclosporine each in 3 patients. Overall, treatment predominantly consisted of aspirin combined with heparin, with hydroxychloroquine and glucocorticoids added as needed (Table 4).

**Table 4:** Treatment and reclassification results (*n* = 45).

Item	All (*n* = 45)	Cases	Notes
Treatment			
Low-dose aspirin	23 (51.1%)	—	
Heparins	34 (75.6%)	—	Low-molecular-weight heparin or fondaparinux
Hydroxychloroquine	29 (64.4%)	—	
Glucocorticoids	18 (40%)	—	
Immunoglobulin/other immunosuppressants	IVIG: 4/45 (8.9%); cyclophosphamide: 3/45 (6.7%); cyclosporine: 3/45 (6.7%)	—	IVIG 4; cyclophosphamide 3; cyclosporine 3
2023 ACR/EULAR reclassification (laboratory-domain analysis)			
Definitively met the ≥ 3-point laboratory threshold	7/41 (17.1%)	P3, P4, P20, P24, P29, P33, P39	High- or moderate-titer IgG solid-phase positivity
Additional cases meeting the threshold under upper-bound assumptions	Up to 4 more	P1, P7, P28, P32	P1, P28 requires a quantitative titer ≥ 40 units and persistent positivity; P7, P32 requires persistent LAC positivity
Primary evaluable-case result	7/41 (17.1%) to 11/41 (26.8%)	—	41 patients had evaluable historical serologic documentation
Conservative sensitivity result	7/45 (15.6%) to 11/45 (24.4%)	P10, P17, P34, P42 counted below threshold	Non-assessable cases were included in the denominator and counted as below threshold
Final classification proportion	Not determined	—	Final classification also requires a clinical-domain score ≥ 3 and confirmation of persistent positivity

The primary analysis used 41 evaluable patients. The conservative sensitivity analysis retained all 45 patients and counted P10, P17, P34 and P42 as below the laboratory threshold. IgA and non-criteria antibodies are not scored; a single positive LAC result scores 1 point, whereas persistent positivity scores 5 points.

ACR, American College of Rheumatology; EULAR, European Alliance of Associations for Rheumatology; IgA, immunoglobulin A; IgG, immunoglobulin G; IVIG, intravenous immunoglobulin; LAC, lupus anticoagulant.

### 3.6 2023 ACR/EULAR reclassification

Scoring was performed case by case, with the laboratory domain treated as the key step (only criteria IgG/IgM isotypes were scored; IgA and non-criteria antibodies were excluded; solid-phase results required ≥40 units). Among the 41 patients with evaluable serologic documentation, 7/41 (17.1%) definitively met the ≥3-point laboratory threshold: P3 (high-titer aCL IgG, 102.32; 5 points), P4 (anti-β2GPI IgG, 46.03; 4 points), P20 (high-titer aCL IgG, 162.00; 5 points), P24 (high-titer aCL IgG, 161.81; 5 points), P29 (high-titer aCL IgG, 91.46; 5 points), P33 (anti-β2GPI IgG, 59.04; 4 points), and P39 (high-titer aCL IgG, 150.17; 5 points). Four additional patients met the threshold only under the upper-bound assumptions, producing an upper estimate of 11/41 (26.8%): P1 and P28 required an aCL IgG titer ≥40 units plus persistent positivity, and P7 and P32 required persistent LAC positivity. The remaining 30 evaluable patients (73.2%) were below the laboratory threshold: P2, P5, P6, P8, P9, P14, P16, P18, P21, P23, P25, P26, P30, P36, P40, P41, P43 and P45 had isolated or predominant IgM positivity; P19 and P44 had aCL IgG below the 40-unit threshold (35.21 and 28.19, respectively); P11, P13, P15, P22, P27, P35 and P37 had non-criteria antibodies only; P12, P31 and P38 had only anti-β2GPI IgA. P10, P17, P34 and P42 were not assessable because their historical serologic documentation was incomplete (Table 4).

Because the solid-phase domain requires persistent positivity and most antibody testing in this cohort consisted of a single measurement, the high-weight scores above remain contingent on confirmation of persistence; final classification also requires an independent clinical-domain score of ≥3. Owing to incomplete persistence data, missing quantitative titers, and incomplete details for some obstetric events, an exact final reclassification proportion could not be determined. The primary evaluable-case result was 7/41 (17.1%) definitively meeting the laboratory-domain threshold and 11/41 (26.8%) meeting it under the upper-bound assumptions. In the conservative sensitivity analysis, which counted P10, P17, P34 and P42 below threshold, the corresponding results were 7/45 (15.6%) and 11/45 (24.4%).

### 3.7 Outcomes in patients identified via non-criteria antibodies

To evaluate whether patients carrying non-criteria antiphospholipid antibodies face clinically meaningful obstetric risks despite failing to reach the 2023 ACR/EULAR laboratory threshold, we descriptively examined the subgroup of 14 patients (P6, P8, P9, P11, P13, P15, P21, P22, P25, P27, P30, P35, P37, and P40) with non-criteria antibodies (Supplementary Table S2). Seven patients (P6, P8, P9, P21, P25, P30, and P40) also had low-weighted criteria serology limited to IgM isotypes, whereas seven (P11, P13, P15, P22, P27, P35, and P37) were positive exclusively for non-criteria antibodies, including aPI, aPA, aPE, aPG, aPS/PT, and anti-annexin V.

All 14 patients received antithrombotic treatment, with HCQ added in 10 patients and prednisone added in P11, P22, and P27. Adverse obstetric outcomes nevertheless occurred in six patients: P11 experienced a miscarriage at 9 weeks despite treatment with LDA, LMWH, HCQ, and prednisone; P13 developed pre-eclampsia and FGR and delivered preterm at 35.4 weeks (birth weight, 2,380 g); and P8 delivered preterm at 36.2 weeks with mild FGR (birth weight, 2,580 g); P22 and P35 experienced miscarriages at 10 and 8 weeks, respectively; and P25 developed pre-eclampsia and FGR and delivered preterm at 35.6 weeks (birth weight, 2,240 g). P6, P9, P15, P21, P27, P30, P37, and P40 had live births at 37.5, 38.1, 37.0, 38.0, 37.4, 38.2, 37.6, and 38.4, respectively, without recorded pregnancy complications. These descriptive findings indicate that adverse maternal-fetal events can occur in patients with non-criteria antibodies, but the small subgroup and absence of an untreated comparator preclude causal or treatment-effect inferences. Excluding such patients solely on the basis of the 2023 classification threshold may therefore risk under-recognition in clinical practice.

## 4 Discussion

This cohort was dominated by the obstetric phenotype (primary APS in 43/45 patients; 71.1% with pregnancy loss), whereas positivity for high-weight criteria serology was relatively uncommon (aCL IgG, 20%; anti-β2GPI IgG, 4.4%; LAC, 4.4%). IgM isotypes and non-criteria antibodies were comparatively common. Thus, many patients in this real-world OAPS cohort belonged to serologic groups that receive little or no weight under the new criteria rather than to the high-weight serologic groups emphasized by the classification system.

Although 75.6%–84.4% of patients in this cohort would be difficult to classify under the new criteria, the cohort-wide live-birth rate reached 80% after conventional OAPS anticoagulant and immunomodulatory treatment. This outcome suggests that patients not captured by the new criteria—including those with non-criteria antibodies, IgA isotypes, isolated IgM, or low-titer antibodies—are not necessarily at low risk and may still benefit from current OAPS treatment strategies. Using the 2023 criteria as a diagnostic threshold or as the sole basis for withdrawing treatment could deny appropriate therapy to patients who may otherwise achieve a live birth, thereby increasing the risk of adverse pregnancy outcomes.

The exploratory outcome-stratified comparison did not identify a statistically significant difference in documented standardized pharmacologic intervention between the successful and unsuccessful groups [28/36 (77.8%); Fisher's exact *p* = 0.666]. This null result should not be interpreted as evidence against treatment benefit: only nine patients were in the unsuccessful group, treatment regimens overlapped, a zero cell produced an unstable effect estimate, and confounding by indication is likely because higher-risk patients may have received more intensive therapy.

Given the limitations of this single-center cohort, we compared our results with published obstetric and pregnancy APS reclassification cohorts (Table 5). The predominance of IgM and the relatively high frequency of non-criteria antibodies in our cohort are consistent with reports of non-criteria OAPS, in which adverse pregnancy outcomes may be comparable to those in classic OAPS despite these patients not being fully captured by current criteria (12). A Spanish cohort found that 58% of patients excluded under the 2023 criteria were positive only for isolated IgM anticardiolipin or anti-β2GPI antibodies, the same serologic pattern observed in patients P2, P5, P6, P8, and P9 in our cohort (13).

**Table 5:** Comparison with published obstetric/pregnancy APS reclassification cohorts.

Study/cohort	*n*	Met Sydney criteria	Met 2023 criteria	Main exclusion mechanism
EUROAPS registry (8)	1,200	100% (enrollment)	∼21.3% (256)	Low weight of obstetric domain; IgM; 40-unit cutoff
Two-center Italian cohort (14)	122	75.4%	23%	Low sensitivity; missed future obstetric events
Primary APS pregnancy cohort (13)	93	100%	25.8% (24)	Underrepresentation of obstetric domain and IgM
Chinese APS cohort (10)	436	Sensitivity 98%	Sensitivity 82%	Tightened obstetric definition; isolated IgM
APS Chinese Collaborative cohort—obstetric (9)	427	Sensitivity 93%	Sensitivity 56%	Marked decline in obstetric-phenotype sensitivity
Present study	45	41 evaluable; 4 not assessable	Laboratory domain: 7/41 (17.1%) to 11/41 (26.8%)*	Non-criteria antibodies; IgA; isolated IgM; single-positive LAC; incomplete serology (P10/P17/P34/P42)

APS, antiphospholipid syndrome; IgA, immunoglobulin A; IgM, immunoglobulin M; LAC, lupus anticoagulant.

* The present-study values refer to the laboratory domain among 41 evaluable patients. A conservative sensitivity analysis counting both non-assessable cases below threshold yielded 7/45 (15.6%) to 11/45 (24.4%). Final classification also requires clinical-domain scoring and confirmation of persistent positivity. Cohort metrics are not directly comparable.

For consistency, the primary evaluable-case laboratory-domain result in our cohort was 7/41 (17.1%) to 11/41 (26.8%), whereas the conservative sensitivity result was 7/45 (15.6%) to 11/45 (24.4%). These are laboratory-domain estimates only. Other cohorts report either full-criteria classification proportions or sensitivity against an expert-diagnosis standard; therefore, the metrics are not directly comparable and are presented only to show the consistent loss of the obstetric phenotype under the 2023 criteria. In the EUROAPS registry, the obstetric domain alone excluded 68% of 1,200 patients; combined with insufficient IgM weighting and the 40-unit cutoff, only about 256 patients (21.3%) ultimately remained classifiable (8). In a two-center Italian cohort of 122 patients with primary OAPS, 75.4% met the Sydney criteria versus only 23% the 2023 criteria, and the new criteria failed to identify patients who subsequently developed obstetric complications (14). Among a cohort of 93 pregnant women with primary APS, only 24 (25.8%) met the new criteria, with a marked reduction among patients with early or late fetal loss and IgM-isotype antibodies (13). Chinese data show a similar pattern: a large validation cohort reported an overall sensitivity of 82%, with the decline attributable specifically to the tightened obstetric definition and the exclusion of isolated IgM (10); in the Antiphospholipid Syndrome Chinese Collaborative cohort, the sensitivity for obstetric APS was only 56%, compared with 93% under the Sydney criteria (9). Other Chinese single-center and Mexican cohorts likewise showed high specificity with reduced overall sensitivity (15, 16), and some authors have explicitly cautioned that the new criteria may miss genuine obstetric patients (17). The characteristics of the patients excluded in our cohort—P11 with non-criteria antibodies only, P7 with IgA isotype, and several patients with isolated IgM positivity—align precisely with the mechanisms identified in these studies.

The antibodies that are downweighted or unscored under the new criteria are not clinically irrelevant. Although the IgM isotype is a weaker predictor of thrombosis than IgG, it remains associated with vascular and obstetric phenotypes (18); among non-criteria antibodies, anti-phosphatidylserine/prothrombin (aPS/PT) and anti-prothrombin antibodies have repeatedly been shown to be associated with thrombosis, frequently overlap with lupus anticoagulant activity, and are common in high-risk populations (19–22). These antibodies are receiving increasing attention in the diagnostic evaluation of aPL-related disease (23). A classification system that excludes them will inevitably miss a subset of at-risk obstetric patients.

It should be emphasized that the 2023 criteria were designed to select a highly specific, homogeneous population for research purposes and were not intended as a diagnostic or treatment-decision tool (7, 24). The increase in specificity to 99% came at the cost of a decline in sensitivity to 84%, which is particularly disadvantageous for the obstetric phenotype. For patients who fall outside the new criteria—especially those identified only through non-criteria antibodies—decisions about whether to withdraw or adjust anticoagulant or antiplatelet therapy should be based on the complete antibody profile, prior pregnancy outcomes, and individual risk, informed by current management recommendations and observational treatment evidence (3, 24, 25), rather than on the sole basis that a patient “does not meet the new criteria.”

This study is limited by its single-center, retrospective design and small sample size (*n* = 45). Some antibodies were measured only once, and the persistence and quantitative titer data required for laboratory-domain scoring were incomplete. Although we analyzed live-birth rates and pregnancy complications, larger controlled studies are needed to quantify associations between specific non-criteria antibody subtypes and adverse pregnancy outcomes. The original serologic documentation for P10, P17, P34, and P42 was unavailable, preventing verification of their Sydney laboratory-criterion status and 2023 laboratory-domain score. The primary evaluable-case laboratory-domain result was 7/41 (17.1%) to 11/41(26.8%), whereas the conservative sensitivity result was 7/45 (15.6%) to 11/45 (24.4%), after P10, P17, P34, and P42 were counted below threshold. The value of this study lies in its case-by-case mapping of a real-world OAPS cohort to the new criteria and its transparent handling of missing persistence data. Larger prospective cohorts with antibody retesting and complete obstetric outcome data are required for validation.

Methodological heterogeneity in non-criteria aPL testing is an additional limitation. The assays differed in manufacturer, antigen system, reporting units, and positivity cutoffs, and quantitative values were therefore not treated as directly comparable across platforms. Incomplete lot-specific documentation may also have introduced batch-related variability and potential serostatus misclassification. Accordingly, the prevalence and outcome patterns for individual non-criteria antibodies should be interpreted cautiously and should be validated prospectively using standardized assays and harmonized cutoff definitions.

A further limitation is that we did not perform a within-cohort comparison of adverse pregnancy outcomes (miscarriage, fetal loss, pre-eclampsia) between patients who met and those who did not meet the 2023 criteria. With only seven patients (up to eleven under the upper-bound assumption) reaching the laboratory threshold, the reclassified-in group is too small for any valid between-group statistical testing, and such a comparison would be uninterpretable. Our inference that the excluded patients remain at clinically meaningful risk therefore does not rest on internal comparative data; it is supported instead by external non-criteria OAPS cohorts with comparable adverse-outcome rates and by the adverse pregnancy outcomes documented among the 14 patients with non-criteria antibodies in this cohort. These findings should accordingly be regarded as hypothesis-generating rather than confirmatory.

The evaluable-case laboratory-domain threshold range was 7/41 (17.1%, 95% Wilson confidence interval approximately 8.5%–31.3%) to 11/41 (26.8%, 95% confidence interval approximately 15.7%–41.9%). A conservative sensitivity analysis counting P10, P17, P34, and P42 as below threshold yielded 7/45 (15.6%) to 11/45 (24.4%). We treated the contrast between the evaluable-case and conservative estimates as a sensitivity analysis; a formal subgroup-stratified analysis was not feasible with *n* = 45. As a single-center retrospective series subject to referral and selection bias, and with many antibodies measured only once, these figures are center-specific and cannot be extrapolated to broader obstetric APS populations; they are offered for trend comparison with published cohorts only.

## 5 Conclusion

In this exploratory, single-center retrospective cohort (*n* = 45), many patients were identified through IgM or non-criteria antibodies and did not meet the laboratory-domain threshold of the 2023 criteria. Because of the small sample size and descriptive design, these findings are preliminary and hypothesis-generating rather than confirmatory. They nevertheless suggest that using the new criteria in isolation may leave some patients unrecognized and could contribute to underdiagnosis and undertreatment in the obstetric phenotype. The 2023 criteria may therefore be more appropriate for defining homogeneous research cohorts than as a stand-alone basis for treatment decisions. Whether the obstetric phenotype requires a distinct classification approach must be evaluated in larger, prospective, multicenter studies with long-term follow-up.

## Data Availability

The original contributions presented in the study are included in the article/Supplementary Material, further inquiries can be directed to the corresponding author/s.
