# Supplementary material for: Reclassification of obstetric antiphospholipid syndrome under the 2023 ACR/EULAR criteria: risk of under-recognition in patients with non-criteria antibodies and clinical implications
Source: Front Med (Lausanne). 2026 Oct 2;13:1929786. doi: 10.3389/fmed.2026.1929786 (PMC13631859; doi:10.3389/fmed.2026.1929786)
Supplement: Supplementary file 1 [file Table1.docx]

**Supplementary Table S1. Non-criteria antibody assay platforms and manufacturer-recommended positivity cutoffs**

| **Antibody** | **Manufacturer / platform** | **Isotype** | **Manufacturer- recommended cutoff** |
| --- | --- | --- | --- |
| Anti-phosphatidic acid (aPA) | EUROIMMUN AG (Lübeck, Germany) | IgG / IgM | ≥ 12.0 RU/mL |
| Anti-phosphatidylethanolamine (aPE) | INOVA Diagnostics (San Diego, CA, USA) | IgG / IgM | ≥ 20.0 APL/GPL |
| Anti-phosphatidylglycerol (aPG) | EUROIMMUN AG (Lübeck, Germany) | IgG / IgM | ≥ 12.0 RU/mL |
| Anti-phosphatidylinositol (aPI) | EUROIMMUN AG (Lübeck, Germany) | IgG / IgM | ≥ 12.0 RU/mL |
| Anti-annexin V | INOVA Diagnostics (San Diego, CA, USA) | IgG / IgM | ≥ 20.0 U/mL |
| aPS/PT antibody | INOVA Diagnostics (San Diego, CA, USA) | IgG / IgM | ≥ 30.0 units |

Notes: All assays were performed according to the respective manufacturers' instructions. Positivity was defined using the assay-specific manufacturer-recommended cutoff; cutoffs and reporting units were therefore not uniform across platforms. Lot-specific information and between-lot quality-control records were not available for all retrospective results, so batch-to-batch variation could not be quantified.

Abbreviations: aPA, anti-phosphatidic acid; aPE, anti-phosphatidylethanolamine; aPG, anti-phosphatidylglycerol; aPI, anti-phosphatidylinositol; aPS/PT, anti-phosphatidylserine/prothrombin.
