# Supplementary material for: Reclassification of obstetric antiphospholipid syndrome under the 2023 ACR/EULAR criteria: risk of under-recognition in patients with non-criteria antibodies and clinical implications
Source: Front Med (Lausanne). 2026 Oct 2;13:1929786. doi: 10.3389/fmed.2026.1929786 (PMC13631859; doi:10.3389/fmed.2026.1929786)
Supplement: Supplementary file 2 [file Table2.docx]

**Supplementary Table S2. Clinical characteristics, treatments, and pregnancy outcomes of 14 patients with non-criteria antibodies**

| **Patient ID** | **Non-criteria antibody profile** | **Concomitant criteria aPL*** | **Treatment** | **Pregnancy outcome** | **Gestational age / birth weight** | **Pregnancy complications** |
| --- | --- | --- | --- | --- | --- | --- |
| P6 | aPI; anti-annexin V | Anti-beta2GPI IgM | LDA + LMWH + HCQ | Live birth | 37.5 weeks / 3,050 g | None |
| P8 | aPA; aPE | aCL IgM + anti-beta2GPI IgM | LDA + LMWH + HCQ | Live birth | 36.2 weeks / 2,580 g | Preterm birth; mild FGR |
| P9 | aPG | aCL IgM | LDA + LMWH | Live birth | 38.1 weeks / 3,120 g | None |
| P11 | aPS/PT IgG/IgM; anti-annexin V | None (non-criteria antibodies only) | LDA + LMWH + HCQ + prednisone | Miscarriage | N/A | Early pregnancy loss (9 weeks) |
| P13 | aPE; aPA | None (non-criteria antibodies only) | LDA + LMWH + HCQ | Live birth | 35.4 weeks / 2,380 g | Pre-eclampsia; preterm birth; FGR |
| P15 | aPS/PT IgG | None (non-criteria antibodies only) | LDA + LMWH | Live birth | 37.0 weeks / 2,850 g | None |
| P21 | aPA; aPG | aCL IgM + anti-beta2GPI IgM | LDA + LMWH + HCQ | Live birth | 38.0 weeks / 3,010 g | None |
| P22 | aPS/PT IgG | None (non-criteria antibodies only) | LDA + LMWH + HCQ + prednisone | Miscarriage | N/A | Early pregnancy loss (10 weeks) |
| P25 | aPE; anti-annexin V | aCL IgM + anti-beta2GPI IgM | LDA + LMWH + HCQ | Live birth | 35.6 weeks / 2,240 g | Pre-eclampsia; preterm birth; FGR |
| P27 | aPA; aPE | None (non-criteria antibodies only) | LMWH + HCQ + prednisone | Live birth | 37.4 weeks / 2,740 g | None |
| P30 | aPI | aCL IgM + anti-beta2GPI IgM | LDA + LMWH + HCQ | Live birth | 38.2 weeks / 3,180 g | None |
| P35 | Anti-annexin V | None (non-criteria antibodies only) | LDA + LMWH | Miscarriage | N/A | Early pregnancy loss (8 weeks) |
| P37 | aPS/PT IgM; aPE | None (non-criteria antibodies only) | LDA + LMWH + HCQ | Live birth | 37.6 weeks / 2,810 g | None |
| P40 | aPG; aPI | aCL IgM + anti-beta2GPI IgM | LMWH | Live birth | 38.4 weeks / 3,220 g | None |

Notes: *Concomitant criteria aPL denotes antibodies included in the 2006 Sydney criteria. Under the 2023 ACR/EULAR criteria, isolated IgM positivity scores 1 point and does not reach the laboratory-domain threshold (≥ 3 points).

Abbreviations: aCL, anticardiolipin; anti-β2GPI, anti-β2-glycoprotein I; aPA, anti-phosphatidic acid; aPE, anti-phosphatidylethanolamine; aPG, anti-phosphatidylglycerol; aPI, anti-phosphatidylinositol; aPL, antiphospholipid antibody; aPS/PT, anti-phosphatidylserine/prothrombin; FGR, fetal growth restriction; HCQ, hydroxychloroquine; LDA, low-dose aspirin; LMWH, low-molecular-weight heparin.
